# Health Disparities Among Children Who Are HIV-Exposed but Without HIV: A Narrative Literature Review and Call to Action for Further Research

**DOI:** 10.7759/cureus.99628

**Published:** 2025-12-19

**Authors:** Amy Parnell, Elisa Lopez, Michelle Moorhouse, Cecelia Hutto, Kathleen M Powis, Ana M Puga

**Affiliations:** 1 Global Medical Affairs, ViiV Healthcare, Brentford, GBR; 2 Global Medical Affairs, ViiV Healthcare, Madrid, ESP; 3 Global Medical Affairs, ViiV Healthcare, Johannesburg, ZAF; 4 Pediatric Infectious Diseases, University of Alabama at Birmingham, Birmingham, USA; 5 Internal Medicine and Pediatrics, Immunology and Infectious Diseases, Massachusetts General Hospital, Harvard T.H. Chan School of Public Health, Boston, USA; 6 Global Pediatrics, ViiV Healthcare, Durham, USA

**Keywords:** hiv-exposed uninfected, immune dysfunction, infant growth, morbidity, neurodevelopmental outcomes

## Abstract

Children who are HIV-exposed but without HIV (HEU) face increased health risks compared with children without HIV exposure, including risk of higher preterm delivery, infectious morbidities, mortality, immune dysfunction, poorer growth, and neurodevelopmental delays. Underlying mechanisms are not fully understood but likely involve multiple intersecting factors, including HIV-induced parental inflammation, altered immune function, in utero antiretroviral therapy exposure, suboptimal breast/chestfeeding, and socioeconomic disparities. Although some differences appear transient, others may persist and predispose children who are HEU to a higher risk of cardiovascular, metabolic, pulmonary, and psychiatric disorders later in life. Dedicated longitudinal studies are needed to further characterize the etiologies and long-term clinical implications of these health disparities, devise tools to identify factors associated with poorer outcomes, and evaluate clinical management strategies and interventions to optimize outcomes in this growing population.

## Introduction and background

Approximately 1.3 million people with HIV become pregnant each year [1]. It is critical that these individuals receive antiretroviral therapy (ART) to reduce the risk of perinatal HIV acquisition (during pregnancy, childbirth, and breast/chestfeeding), which is as high as 40% without ART and <1% with sustained viral suppression through the perinatal period [2,3]. Due to investments in reducing pediatric HIV acquisition [1], ART coverage in pregnancy has risen from no coverage to over 80% since 2000 [2,4]. A result of this success is that HIV acquisition among children aged <15 years has decreased by ~77%; subsequently, the population of children who are HIV-exposed but without HIV (HEU) has grown to ~16.1 million as of 2023 [5].

Despite success in preventing vertical transmission, children who are HEU face considerable health challenges, including worsened growth, neurodevelopmental delays, and immune dysfunction, relative to children without HIV exposure [6-9]. Consequently, they also experience increased risks of mortality, cardiometabolic perturbations, depression, anxiety, and hospitalization from infectious diseases [10-13]. Identifying the causes of these disparities is essential to inform targeted interventions. However, the mechanisms underlying these health challenges are complex and most likely involve multiple intersecting biological, social, and structural factors, requiring further elucidation [7].

Global health organizations have established goals to ensure that all individuals affected by HIV, including children, survive and thrive to live long and healthy lives [14-16]. For instance, goal 3 of the United Nations’ Sustainable Development Goals and the response to HIV/AIDS aim to “ensure healthy lives and promote well-being for all at all ages” [14,15]. Likewise, the Global Alliance to End AIDS in Children, which launched in 2022, works to guarantee high-quality care for children affected by HIV [16]. To advance these global commitments, it is essential to address the health disparities experienced by children who are HEU.

One challenge to improving the health of children who are HEU is the difficulty in interpreting the existing evidence. Although studies increasingly document adverse health outcomes in this population, they are often limited by inconsistent methodological rigor, including secondary use of data, small sample sizes, and limitations in study designs [7,9]. Understanding current findings can help ensure that children who are HEU receive the necessary support as they mature. To this end, we performed a narrative literature review shaped by targeted literature reviews and expert recommendations from the authors (see Appendices for details of the literature search strategy) to review the development, immunologic health, and morbidities of children who are HEU and highlight the need for more robust research and effective interventions for this growing population.

## Review

Growth and development

The impact of HIV exposure on growth is not fully understood, with conflicting study results (Table 1). Low birth weight is a key determinant of mortality and stunting (i.e., impaired linear growth) in children, and indicates reduced human capital in adulthood [7]. Before universal ART for pregnant persons, children who are HEU were more likely to have low birth weight (1.7-fold) or stunting (1.2-fold) compared with children without HIV exposure [17,18]. Recent studies on ART use during pregnancy continue to report increased risk of growth deficits among children who are HEU [19-29], including higher likelihood of low birth weight (1.6- to 3.9-fold) [19,20], preterm delivery (1.2-fold) [19], small for gestational age (1.3-fold) [19], and stunting (1.6- to 3.4-fold) [20,22-24], suggesting that poor linear growth is influenced by the in utero milieu and persists during childhood [7]. While this suggests fetal programming as an etiology of growth outcomes, not all studies in which ART was used during pregnancy have reported birth outcomes or growth differences (Poster: Kgole SW, Jao J, Sun S, et al. Similar Birth Anthropometrics With in Utero Exposure to Dolutegravir or Efavirenz. Conference on Retroviruses and Opportunistic Infections 2019; 2019; Poster: King’e M, Njuguna I, McGrath CJ, et al. Similar Early Growth in HEU and HUU Infants With Maternal ART Optimisation. Conference on Retroviruses and Opportunistic Infections 2022; 2022; Poster: Powis KM, Dunk C, Mmasa K, et al. Pregnancy Hormonal Dysregulation Correlates With HIV-Exposed Infant Growth Outcomes. Conference on Retroviruses and Opportunistic Infections 2023; 2023) [30,31]. Notably, among studies where birth outcomes and/or growth were comparable between children who are HEU and those without HIV exposure, none reported sample size calculations, so it is unclear if they were adequately powered to detect a clinically meaningful difference.

**Table 1:** Summary of Growth and Development Findings in Children Who Are HEU ART, antiretroviral therapy; BAZ, BMI-for-age *z* score; BMIZ, body mass index *z* score; cART, combination ART; d4T, stavudine; DTG, dolutegravir; EFV, efavirenz; FTC, emtricitabine; HAZ, height-for-age *z* score; HCAZ, head circumference-for-age *z* score; HEU, HIV-exposed but without HIV; IQR, interquartile range; LAZ, length-for-age *z* score; LPV, lopinavir; MUACZ, mid-upper arm circumference *z* score; NNRTI, non-nucleoside reverse transcriptase inhibitor; NR, not reported; NRTI, nucleoside reverse transcriptase inhibitor; NVP, nevirapine; OR, odds ratio; PI, protease inhibitor; RTV, ritonavir; sdNVP, single-dose NVP; SGA, small for gestational age; 3TC, lamivudine; TDF, tenofovir disoproxil fumarate; WAZ, weight-for-age *z* score; WHZ, weight-for-height *z* score; WLZ, weight-for-length *z* score; XTC, lamivudine or emtricitabine; ZDV, zidovudine. ^a^Number of children who are HEU.

Reference	Country/region	Study period (y)	Age	N^a^	Parental ART exposure	Parameters assessed	Key findings relevant to children who are HEU
Awadu et al., 2023 [32]	Uganda	2017-2019	6-18 years	242	Intrapartum sdNVP ± ZDV (17%) or sdNVP + ZDV + 3TC (20%), in utero cART (21%), none (43%)	HAZ	No significant difference in HAZ (mean difference, -0.06; 95% CI, -0.23 to 0.14) vs. children without HIV exposure. HAZ was similar with sdNVP ± ZDV (-0.16; 95% CI, -0.46 to 0.14) or sdNVP + ZDV + 3TC exposure (0.08; 95% CI, -0.20 to 0.35), lower with no ART exposure (-0.27; 95% CI, -0.52 to 0.00), and higher with cART exposure (0.41; 95% CI, 0.10 to 0.71) vs. children without HIV exposure.
Dara et al., 2018 [25]	United States	2008-2012	Birth	155	PI-based (66%), non-PI-based (15%), unknown (19%)	Birth weight	Significantly lower birth weight vs. infants without HIV exposure (mean ± SD, 2972 ± 616 vs. 3167 ± 644 g; P < 0.01). No difference in mean birth weight among infants who are HEU with PI-based vs. non-PI-based ART exposure (2958 vs. 2976 g; P = 0.91).
du Toit et al., 2024 [26]	South Africa	2017-2023	Birth to 24 months	29	TDF/FTC + EFV	WHZ, BAZ, MUACZ	Lower median WHZ at 6 weeks (-0.5; IQR, -1.4 to 0.6 vs. 0.4; IQR, -0.3 to 1.7; P = 0.012) and 10 weeks (-0.4; IQR, -1.2 to 0.8 vs. 0.6; IQR, -0.6 to 1.4; P = 0.019). Lower BAZ at 6 weeks (-0.5; IQR, -1.2 to 0.5 vs. 0.7; IQR, -0.2 to 1.1; P = 0.01) and 10 weeks (-0.5; IQR, -1.0 to 0.2 vs. 0.7; IQR, -0.6 to 1.0; P = 0.005). Lower MUACZ at 6 months (0.2; IQR, -0.2 to 0.7 vs. 0.7; IQR, 0.2 to 1.5; P = 0.038) vs. infants without HIV exposure.
Floridia et al., 2023 [21]	Malawi	2019-2021	Birth to 1 year	163	TDF/3TC/EFV (76%), TDF/3TC/DTG (8%), other (16%)	BMIZ, WLZ, stunting, underweight	Significantly lower BMIZ (mean ± SD, 0.69 ± 1.36 vs. 1.31 ± 1.70; P = 0.010) and WLZ (1.06 ± 1.50 vs. 1.94 ± 1.58; P = 0.001) at 6 weeks but not at 6 or 12 months vs. infants without HIV exposure. No significant differences in rate of stunting or being underweight at 6 weeks (stunting: 18% vs. 26%; P = 0.195; underweight: 5% vs. 6%; P = 0.762), 6 months (stunting: 20% vs. 20%; P = 0.877; underweight: 3% vs. 0%; P = 0.998), or 12 months (stunting: 23% vs. 25%; P = 0.790; underweight: 8% vs. 4%; P = 0.513) vs. infants without HIV exposure.
Fowler et al., 2022 [23]	Malawi and Uganda	2013-2014	12-60 months	Malawi, N = 238; Uganda, N = 240	ZDV (45%-47%), cART (53%-55%)	HAZ, WAZ, stunting	Adjusted mean HAZ (mean difference, 60 months, -0.25; 95% CI, -0.46 to -0.04; P = 0.020) and WAZ (48 months, -0.18; 95% CI, -0.35 to -0.001; P = 0.050) scores were significantly lower at all time points through 48-60 months vs. children without HIV exposure in Uganda. Stunting rates were significantly higher at 12 months (adjusted OR, 3.38; 95% CI, 1.56 to 7.31), 24 months (1.99; 95% CI, 1.00 to 3.98), and 48-60 months vs. children without HIV exposure in Uganda. No significant differences in growth measures or stunting rates were observed between groups in Malawi.
Jumare et al., 2019 [20]	Nigeria	2013-2017	Birth to 18 months	307	cART per Nigerian guidelines	WAZ, LAZ, WLZ, BAZ, stunting, underweight	Significantly lower WAZ (mean difference, -0.28; 95% CI, -0.47 to -0.09; P = 0.003), LAZ (-0.35; 95% CI, -0.57 to -0.12; P = 0.002), WLZ (-0.3; 95% CI, -0.52 to -0.09; P = 0.006), and BAZ (-0.42; 95% CI, -0.63 to -0.21; P < 0.001). Higher odds of stunting (OR, 1.55; 95% CI, 1.12 to 2.15; P = 0.009) and being underweight (2.4; 95% CI, 1.41 to 4.09; P = 0.001) vs. children without HIV exposure.
Kgole et al., 2019 (Poster: Kgole, 2019)	Botswana	NR	16 weeks gestation to 3 days post-delivery	275	DTG/TDF/FTC (57%), EFV/TDF/FTC (43%)	WAZ, LAZ	No significant differences in birth WAZ (mean, -0.13; 95% CI, -0.25 to -0.01 vs. 0.00; 95% CI, -0.16 to 0.16; P = 0.20) or LAZ (1.07; 95% CI, 0.87 to 1.26 vs. 1.17; 95% CI, 0.93 to 1.41; P = 0.51) vs. infants without HIV exposure. No significant differences in birth WAZ (-0.09; 95% CI, -0.26 to 0.09 vs. -0.18; 95% CI, -0.36 to 0.00; P = 0.45) or LAZ (1.16; 95% CI, 0.89 to 1.43 vs. 0.95; 95% CI, 0.66 to 1.23; P = 0.28) among infants who are HEU with in utero DTG- vs. EFV-based ART exposure.
King’e et al., 2022 (Poster: King’e, 2022)	Kenya	2021	4-10 weeks	365	DTG-based (62%), EFV-based (29%), PI-based or other (9%)	WAZ, LAZ, WLZ	Similar median WAZ (0.13; IQR, 4.50 vs. 0.02; IQR, 1.12; P = 0.52), LAZ (-0.55; IQR, 3.31 vs. -0.48; IQR, 1.44; P = 0.61), and WLZ (1.10; IQR, 7.13 vs. 0.67; IQR, 1.25; P = 0.11) vs. infants without HIV exposure. No growth differences were observed in relation to timing of parental ART initiation, parental ART regimen, or parental viral load among infants who are HEU.
Le Roux et al., 2019 [22]	South Africa	2013-2016	Birth to 12 months	461	EFV + FTC + TDF	WAZ, LAZ, stunting	WAZ was consistently lower from 6 weeks to 12 month (crude overall β, -0.34; 95% CI, -0.47 to -0.21). LAZ was significantly lower at 12 month (crude overall β, -0.43; 95% CI, -0.61 to -0.25), and odds of being stunted were higher at 12 month (OR, 2.67; 95% CI, 1.41 to 5.06) vs. children without HIV exposure.
le Roux et al., 2023 [33]	South Africa	2013-2016	Birth to 12 months	461	EFV-based	WAZ, underweight	Having both parental HIV and household food insecurity was significantly associated with lower WAZ (β, -0.43; 95% CI, -0.61 to -0.25; P < 0.0001) and increased odds of being underweight (OR, 3.90; 95% CI, 1.67 to 9.11; P = 0.002) vs. children without HIV exposure from food secure households.
Locks et al., 2017 [17]	Tanzania	2004-2007	6 weeks to 18 month	2088	NVP prophylaxis, d4T + 3TC + NVP in accordance with national guidelines	HAZ, WAZ, WHZ, stunting, underweight	At 6 weeks, lower mean HAZ, WAZ, and WHZ and significantly higher risk of being underweight (adjusted relative risk, 1.90; 95% CI, 1.33 to 2.73; P < 0.001) vs. children without HIV exposure. From 6 weeks to 18 month, significantly lower rates of stunting vs. children without HIV exposure (adjusted hazard ratio, 0.64; 95% CI, 0.51 to 0.81; P < 0.001).
Neary et al., 2022 [24]	Kenya	2013	6 weeks to 9 months	456	cART (68%), ZDV (18%), none (14%)	WAZ, LAZ, HCAZ	Mean WAZ was significantly lower at 6 weeks (-0.41; 95% CI, -0.58 to -0.24 vs. -0.09; 95% CI, -0.19 to 0.01; P < 0.001) but not at 9 month (-0.38; 95% CI, -0.55 to -0.21 vs. -0.21; 95% CI, -0.33 to -0.10; P = 0.10) vs. children without HIV exposure. Mean LAZ was significantly lower at 6 weeks (-0.99; 95% CI, -1.39 to -0.58 vs. -0.31; 95% CI, -0.55 to -0.07; P = 0.001) and 9 month (-0.60; 95% CI, -0.94 to -0.26 vs. -0.07; 95% CI, -0.25 to 0.12; P = 0.005), and stunting prevalence was higher at 6 weeks (34% vs. 18%; P < 0.001) and 9 month (20% vs. 10%; P < 0.001) vs. children without HIV exposure. Mean HCAZ was similar at 6 weeks (0.56; 95% CI, 0.30 to 0.81 vs. 0.77; 95% CI, 0.63 to 0.92; P = 0.11) but lower at 9 month (-0.10; 95% CI, -0.52 to 0.31 vs. 0.41; 95% CI, 0.26 to 0.56; P = 0.018) vs. children without HIV exposure.
Nyemba et al., 2022 [27]	South Africa and Zambia	2017-2018	≥6 weeks to 6 months	395	TDF + XTC + EFV (80%), other (17%)	WAZ, WLZ, LAZ	Lower mean ± SD WAZ and WLZ at 6-10 weeks (WAZ, -0.34 ± 1.14 vs. 0.05 ± 1.16; P < 0.001; WLZ, 0.96 ± 1.76 vs. 1.43 ± 1.78; P < 0.001) and 6 month (WAZ, -0.10 ± 1.24 vs. 0.17 ± 1.24; P = 0.002; LAZ, -1.12 ± 1.53 vs. -0.76 ± 1.48; P = 0.001) vs. infants without HIV exposure. Mean ± SD WAZ (6-10 weeks, -0.34 ± 1.15 vs. -0.35 ± 1.13; P = 0.92; 6 month, -0.07 ± 1.27 vs. -0.16 ± 1.19; P = 0.48), LAZ (6-10 weeks, -1.16 ± 1.32 vs. -1.04 ± 1.46; P = 0.37; 6 month, -1.04 ± 1.58 vs. -1.26 ± 1.41; P = 0.17), and WLZ (6-10 weeks, 1.01 ± 1.69 vs. 0.87 ± 1.86; P = 0.43; 6 month, 0.90 ± 1.48 vs. 0.94 ± 1.39; P = 0.78) were similar among infants who are HEU with ART exposure from time of conception vs. those exposed later in gestation.
Powis et al., 2023 (Poster: Powis, 2023)	Botswana	NR	Birth to 1 year	39	DTG/TDF/FTC	WAZ, LAZ	At birth, WAZ (mean, -0.26; 95% CI, -1.17 to 0.95 vs. 0.03; 95% CI, -0.96 to 0.48; P = 0.72) and LAZ (1.12; 95% CI, 0.47 to 2.06 vs. 1.12; 95% CI, -0.10 to 2.67; P = 0.72) were similar vs. infants without HIV exposure.
Ramokolo et al., 2017 [19]	South Africa	2012-2013	4-8 weeks	2599	Pre-conception ART (24%), post-conception ART (30%), ZDV (34%), or none (13%)	Low birth weight, SGA, underweight	Higher odds of low birth weight (adjusted OR, 1.6; 95% CI, 1.3 to 1.9; P < 0.01), SGA (1.3; 95% CI, 1.1 to 1.6; P < 0.01), and being underweight for age (1.5; 95% CI, 1.2 to 1.8; P < 0.01) vs. infants without HIV exposure. Among infants who are HEU, odds of having low birth weight or being SGA or underweight for age were similar with pre-conception ART (low birth weight, 0.9; 95% CI, 0.6 to 1.3; P = 0.54; SGA, 0.9; 95% CI, 0.6 to 1.3; P = 0.52; underweight for age, 1.1; 95% CI, 0.7 to 1.6; P = 0.78), ZDV (low birth weight, 0.8; 95% CI, 0.6 to 1.1; P = 0.14; SGA, 0.7; 95% CI, 0.5 to 1.0; P = 0.05; underweight for age, 1.1; 95% CI, 0.8 to 1.6; P = 0.64), or no ART exposure (low birth weight, 1.1; 95% CI, 0.8 to 1.6; P = 0.47; SGA, 0.7; 95% CI, 0.4 to 1.1; P = 0.08; underweight for age, 1.4; 95% CI, 0.9 to 2.2; P = 0.12) vs. post-conception ART exposure.
Ray et al., 2024 [30]	Kenya	2011-2016	Birth to 24 months	85	NVP or LPV/RTV with 3TC + ZDV or 3TC + TDF	Birth weight, length, head circumference	There was no observed difference in mean ± SD birth weight (3.2 ± 0.52 vs. 3.2 ± 0.51 kg; P = 0.68), length (47.5 ± 2.69 vs. 47.7 ± 3.08 cm; P = 0.53), or head circumference (34.6 ± 1.97 vs. 35.1 ± 1.86 cm; P = 0.07) or growth curve trajectories for weight, length, and head circumference from birth to 24 month vs. children without HIV exposure.
Rickman et al., 2023 [29]	Kenya	2014-2017	Birth to 23 months	149	EFV (62%), NVP (35%), LPV + RTV (3%), ZDV (1%)	WAZ, LAZ, HCAZ	More likely to belong to smaller growth trajectory classes for WAZ (relative risk ratio, 4.2; 95% CI, 1.9 to 9.3), LAZ (3.3; 95% CI, 1.5 to 7.4), and HCAZ (4.4; 95% CI, 2.1 to 9.1) vs. children without HIV exposure.
Toledo et al., 2022 [28]	Malawi	2014-2018	1-6 to 24 months	1185	TDF + 3TC + EFV	WAZ, LAZ	Data reported in children who are HEU over time (no control group). Median WAZ (-0.64; IQR, -1.52 to 0.17) and LAZ (-2.10; IQR, -3.32 to -1.02) were below the reference median at 1-6 month and demonstrated growth faltering after 12 and 13 month, respectively. No differences in early growth trajectories were observed by timing of parental ART initiation through 24 month.
Wedderburn et al., 2024 [31]	South Africa	2012-2015	Birth to 2 years	247	ZDV (15%), first line (NNRTI + 2 NRTIs, 80%), second line (PI-containing, 5.4%)	Gestational age, birth weight	No statistically significant differences in median gestational age at delivery (39; IQR, 37 to 40 vs. 39; IQR, 38 to 40 weeks; P = 0.2), birth weight (3.08; IQR, 2.66 to 3.36 vs. 3.10; IQR, 2.72 to 3.43 kg; P = 0.4), and low birth weight (14% vs. 15%; P = 0.8) were observed vs. infants without HIV exposure.

Timing and duration of fetal antiretroviral (ARV) exposure and exposure to specific ARVs potentially affect growth in children who are HEU. Studies generally show no differences in growth between children who are HEU exposed to older ART agents, primarily efavirenz (EFV), or nevirapine-based regimens or zidovudine, from conception compared with later in gestation [19,27,28]. Given multidrug ART regimens, it is difficult to disentangle associations between individual ARVs and growth. In a meta-analysis, protease inhibitor-based regimens were associated with preterm delivery [34], exacerbating the risk of poorer growth [35]. DTG-based regimens are associated with a lower prevalence of preterm birth compared with EFV-based regimens [36]. Similarly, infants with in utero DTG exposure have similar birth weights compared with infants without HIV exposure (Poster: Kgole, 2019; Poster: King’e, 2022; Poster: Powis, 2023). Socioeconomic factors may also influence growth, as food insecurity is significantly associated with reduced growth in children who are HEU [33], and suboptimal socioeconomic factors tend to be more prevalent in families affected by HIV [37].

Data on long-term growth outcomes in children who are HEU are limited. Early growth differences in children who are HEU with exposure to three-drug ART regimens appear to be transient, with lower growth metrics observed at 6 to 10 weeks of age relative to children without HIV exposure, but this difference was not observed by 6 months of age [21, 27]. Children who are HEU aged 11 to 18 years with fetal ARV exposure had similar growth to children without HIV exposure, whereas children who are HEU but without fetal ARV exposure exhibited growth deficits [32]. Another study showed an association between higher parental viral load and earlier onset of puberty (Poster: Serghides L, Jacoboson DL, Lee J, et al. Evaluating Associations Between in Utero HIV/ART Exposure and Pubertal Status. Conference on Retroviruses and Opportunistic Infections 2023; 2023).

Overall, available data suggest that ART exposure timing and regimen composition may contribute to growth outcomes in children who are HEU. However, further research is needed to better understand the mechanisms underlying reported differences, which may enable identification of viable interventions to ameliorate differences, such as optimizing parental and child nutrition [22,29]. Importantly, globally harmonized surveillance systems should exist to evaluate growth as newer treatment options, including both traditional ARVs and broadly neutralizing antibodies, become available for pregnant and postpartum persons.

Neurodevelopment

In a meta-analysis exploring neurodevelopmental outcomes in children who are HEU aged zero to five years, most studies found developmental deficits compared with children without HIV exposure, particularly in language and motor function, even with adjustment for socioeconomic, demographic, and health-related factors [6], consistent with more recent publications [38,39]. Imaging studies have demonstrated that newborns and children who are HEU aged up to 11 years had decreased brain volumes, including total and subcortical gray matter structures; neurometabolic profiles indicative of neuroinflammatory processes; and distinct neurometabolic patterns correlating with delayed motor function [40-42]. In two studies, children who were HEU aged nine and 11 years exhibited reductions in brain metabolites indicative of possible neuronal damage [43,44]. Children who are HEU aged seven to 15 years in Botswana were significantly more likely to have lower academic performance overall and in mathematics, science, and English than children without HIV exposure [45]. However, cognitive assessments among school-age children (five to 11 or six to 18 years) were generally comparable between populations [46-48].

The etiology of neurodevelopmental deficits in children who are HEU requires further investigation, as socioeconomic factors do not fully explain these differences. Proposed factors include the in utero inflammatory environment and fetal ARV exposure. In one study, immune dysregulation was demonstrated in pregnant individuals with HIV and in their children who are HEU; decreased levels of immune biomarkers at six to 10 weeks of age were associated with poorer motor development at ~2 years [49]. Studies have examined the association between fetal ARV exposure and risk of developmental delays or neurologic disorders [50-53]. In utero exposure to nucleoside reverse transcriptase inhibitors may be associated with language impairment in children aged three to five years [54]. Early findings for DTG have suggested higher gross motor scores in infants and similar brain volume in children who are HEU aged three to four years with in utero exposure to DTG-based ART versus EFV-based ART (Poster: Bradford LE, Ringshaw JE, Wedderburn CJ, et al. Brain Structure of South African HEU Children Exposed to Dolutegravir Versus Efavirenz. Conference on Retroviruses and Opportunistic Infections 2024; 2024) [55]. Although research in this area is limited, bacterial and viral coinfections could also potentially impact neurodevelopmental outcomes in children who are HEU. For instance, congenital cytomegalovirus infection is the primary cause of non-genetic hearing loss in children, and higher human cytomegalovirus loads are more prevalent in infants who are HEU relative to infants without HIV exposure [56]. 

Given the evolving landscape of treatment options for pregnant and postpartum persons, harmonized study designs and/or surveillance systems are needed to monitor neurodevelopmental outcomes, identify children at risk of poor neurodevelopmental outcomes, and test interventions to improve outcomes. Such studies should include longer-term follow-up to understand academic achievement and incorporate approaches to quantify impact on human capital as this population ages into adulthood.

Immune health and function

**Figure 1:**
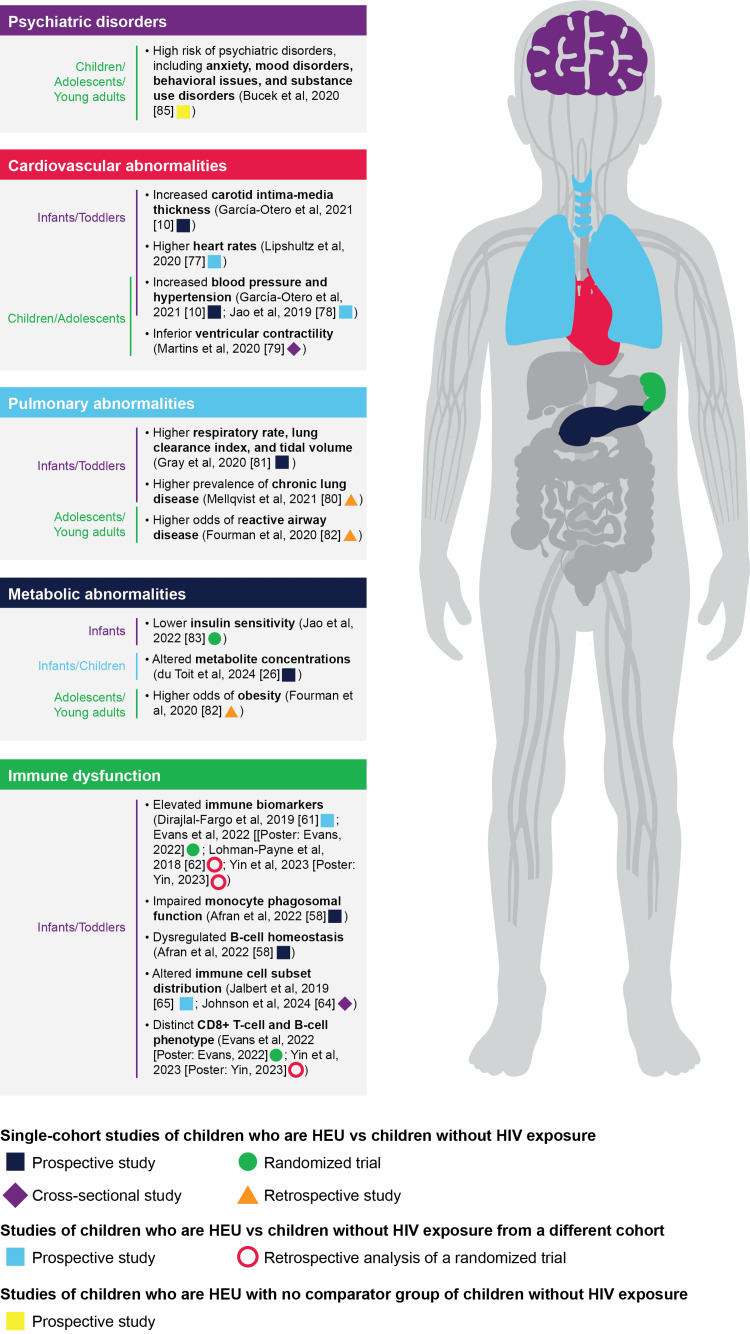
Summary of Findings on Immune Dysfunction and Morbidities in Children Who Are HIV-Exposed but Without HIV Results summarized from the following references: [10,26,58,61,62,64,65,67-74].

Early in life, the innate immune system is the first line of defense against pathogens, with limited adaptive function [57]. Infants who are HEU demonstrate altered immune status compared with infants without HIV exposure, including elevated immune biomarkers (Table 2), dysregulated monocyte function and B-cell homeostasis, and diminished humoral responses (Figure 1; Poster: Evans C, Chasekwa, Broad, et al. Associations Between Maternal HIV and Infant Immune Activation. Conference on Retroviruses and Opportunistic Infections 2022; 2022; Poster: Yin L, Fischer BM, Venturi GM, et al. HIV Exposed Uninfected (HEU) Infants Display Unique Pro-inflammatory Bioprofiles. Conference on Retroviruses and Opportunistic Infections 2023; 2023) [58-63]. Immune cell subset distributions differ between populations, markedly for antigen-presenting cells and regulatory CD4+ T cells [64, 65]. Further, CD8+ T cells and B cells have increased biomarker expression for activation and development, respectively (Poster: Evans, 2022; Poster: Yin, 2023), and immune cells from infants who are HEU have reduced functionality in vitro [58, 65]. Dysregulated immune function creates vulnerability to infections, which may increase the risk of morbidity or mortality among infants who are HEU [58-60]. Etiologies of observed immune alterations most likely involve “immune remodeling” associated with in utero exposure to HIV, ARVs (timing and duration), inflammatory milieu, other pathogens, and other environmental factors [58,60,63].

**Table 2:** Summary of Immunologic Biomarker Findings in Children Who Are HEU APRIL, a proliferation-inducing ligand; BAFF, B-cell–activating factor; CCL4, chemokine (C-C motif) ligand 4; CXCL9, chemokine (C-X-C motif) ligand 9; GM-CSF, granulocyte-macrophage colony-stimulating factor; HAZ, height-for-age *z* score; HEU, HIV-exposed but without HIV; hsCRP, high-sensitivity C-reactive protein; I-FABP, intestinal fatty acid–binding protein; IFN-γ, interferon gamma; IL-1β, interleukin-1β; IL-1RA, interleukin-1 receptor antagonist; IL-2, interleukin-2; IL-4, interleukin-4; IL-6, interleukin-6; IL-8, interleukin-8; IL-10, interleukin-10; IL-12, interleukin-12; IL-21, interleukin-21; IL-22, interleukin-22; IP-10, interferon-gamma induced protein 10; IQR, interquartile range; LBP, lipopolysaccharide-binding protein; MIP-1β, macrophage inflammatory protein-1β; MMP-9, matrix metalloproteinase-9; NGAL, neutrophil gelatinase-associated lipocalin; NR, not reported; oxLDL, oxidized low density lipoprotein; sCD14, soluble CD14; sCD27, soluble CD27; sCD163, soluble CD163; sTNFR1, soluble tumor necrosis factor receptor 1; TNF-α, tumor necrosis factor alpha. ^a^Number of children who are HEU. ^b^Period of the study that recruited women with HIV. ^c^Includes all biomarkers listed in the biomarkers assessed column. ^d^Age not reported.

Reference	Country/ Region	Study period (y)	N^a^	Age	Biomarkers assessed	Key findings relevant to children who are HEU
Baroncelli et al, 2019 [66]	Malawi	2008-2009	72	1-12 months	sCD14, LBP, I-FABP	Data reported in children who are HEU over time (no control group). Increase from 1-12 months in median sCD14 (1 month, 932.5; IQR, 824.3 to 1167.9 ng/mL; 12 months, 1714.3; IQR, 1327.4 to 2036.9 ng/mL) and LBP (1 month, 11.4; IQR, 6.4 to 14.0 µg/mL; 12 months, 16.6; IQR, 10.3 to 23.3 µg/mL). LBP levels negatively correlated with HAZ scores (r = -0.347; P = 0.005). Infants aged 12 months with gastrointestinal infections had higher levels of I-FABP (1442.0 pg/mL with diarrhea vs. 860.0 pg/mL without diarrhea; P = 0.018).
Dirajlal-Fargo et al, 2019 [61]	Brazil	NR	86	Birth to 6 months	sTNFR1, IL-6, IP-10, oxLDL, sCD14, hsCRP	Higher levels of all inflammation biomarkers at birth and higher mean ± SD IL-6 (0.36 ± 0.57 vs. 0.13 ± 0.51 pg/mL; P < 0.0001), sTNFR1 (3.06 ± 0.11 vs. 2.96 ± 0.13 pg/mL; P < 0.0001), and IP-10 (2.01 ± 0.40 vs. 2.21 ± 0.36 pg/mL; P = 0.001) levels at 6 months vs. infants without HIV exposure. Among infants who are HEU, higher hsCRP (P = 0.04) and IP-10 (P = 0.04) were associated with lower weight at birth and 6 months. Except for IP-10 (P < 0.001), parental biomarker levels were not associated with biomarker levels in infants who are HEU.
Evans et al, 2022 (Poster: Evans, 2022)	Zimbabwe	NR	114	1 month	sCD14	Higher sCD14 vs. infants without HIV exposure (adjusted difference, 0.25 × 10⁶; 95% CI, 0.15 × 10⁶ to 0.35 × 10⁶).
Lohman-Payne et al, 2018 [62]	Kenya	1992-1998^b^	22	Birth	IL-6, IL-8	Cord blood IL-8 and IL-6 were >50-fold and ~25-fold higher, respectively, vs. infants without HIV exposure Parental viral load did not appear to mediate infant IL-8 levels.
Miyamoto et al, 2017 [63]	Brazil	NR	58	Birth to 12 years	IL-4, MIP-1β	Few alterations in biomarkers were observed vs. children without HIV exposure. Median IL-4 levels were higher at 6-12 years (1.0 vs. 0.6 pg/mL; P = 0.043) and median MIP-1β levels were lower at 12 months (23.9 vs. 46.0 pg/mL; P = 0.009) vs. children without HIV exposure.
Sevenoaks et al, 2021 [49]	South Africa	2012-2015	77	6 weeks to 28 months	IL-1β, IL-2, IL-4, IL-6 IL-10, IL-12, IFN-γ, GM-CSF, MMP-9, NGAL	IFN-γ (P = 0.005), IL-1β (P < 0.001), IL-2 (P = 0.004), and IL-4 (P = 0.0013) were lower at 24-28 months vs. children without HIV exposure. Among infants who are HEU, inflammation biomarkers at 6-10 weeks were associated with worsened motor function at 24-48 months.
Yin et al, 2023 (Poster: Yin, 2023)	NR	NR	46	Infants^d^	APRIL, BAFF, IL-21, sCD14, sCD163, sCD27, IL-22, CXCL9, CCL4, TNF-α, IL-10, IL-1RA	Higher levels of biomarkers of B-cell development, immune activation, and immune regulation vs. infants without HIV exposure. Among infants who are HEU, inflammation biomarker levels were independent of parental viral load.

Exposure to the inflammatory milieu in utero may prime the developing innate immune system [60]. Infants who are HEU have lower CD4+ and CD8+ T-cell counts, along with increased activated and memory T cells, compared with children without HIV exposure, and HIV exposure in utero may contribute to HIV-specific cellular memory responses. Immune system priming in children who are HEU is complex, resulting in varied immune dysregulation, which may explain the observed diversity of T-cell responsiveness and inflammatory cytokine profiles. Longitudinal studies are needed to determine if in utero priming affects downstream pathogen susceptibility [60].

HIV-induced inflammation can negatively impact transplacental antibody transfer, with infants who are HEU demonstrating reduced transfer of antibodies to numerous antigens [75]. Despite these reductions, protection levels and antibody titers following primary vaccinations are generally similar [60]. Resilience and durability of vaccination response remain uncertain, with one study showing decreasing immunity to the hepatitis B virus over time among children who are HEU (Poster: Dorval S, Boucoiran I, Renaud C, et al. Inadequate Serologic Response Following Vaccination Against Hepatitis B Virus Among Children Who Were Exposed to HIV. 25th International AIDS Conference; July 22-26, 2024).

The developing immune system of infants who are HEU differs from that of infants without HIV exposure, with limited understanding of its multifactorial etiology [60]. Although this population is at higher risk of early-life infection, their humoral response to vaccination is comparable to that of children without HIV exposure, suggesting other mechanisms, such as impaired innate immunity or qualitative antibody response. Future studies should evaluate challenges posed by variations in enrollment age, parental viral load, and ART exposure to clarify the need for targeted interventions. Interventions being evaluated to improve immunity in children who are HEU include *Bifidobacterium infantis* and human milk oligosaccharide supplementation to optimize the gut microbiome, which is partially responsible for training the infant’s maturing immune system [76,77]. 

Morbidity and mortality

Children who are HEU are at higher risk of all-cause mortality through at least 24 months of age compared with children without HIV exposure [17,78]. This population is also at higher risk of all-cause or infectious-cause hospitalization and may have longer hospital admissions [13,17,31,79,80]. However, antenatal ART greatly attenuates mortality risk [81,82], with one study attributing the absence of ART during pregnancy to nearly half of deaths before 24 months of age in children who are HEU [81]. Poor parental health and survival are key risk factors associated with mortality in children who are HEU [11,81,83]. Initiation of ART before conception or early in pregnancy is associated with a lower risk of infant hospitalization [79,84]. Breast/chestfeeding is crucial for reducing mortality and hospitalization rates among infants who are HEU, particularly in settings where overall infant mortality is high. For instance, in a pooled analysis of 21 clinical studies, the risk of infant mortality increased 12.5-fold following cessation of breast/chestfeeding [81]. In a Uganda-based study, after adjusting for sociodemographic factors, non-breast/chestfed infants with fetal HIV and ART exposure had a 10-fold higher risk of hospitalization compared with breast/chestfed infants without HIV exposure; notably, risk of hospitalization was similar between breast/chestfed infants who are HEU and breast/chestfed infants without HIV exposure [85].

Other health disparities have been noted (Figure 1). For example, several studies have identified cardiovascular abnormalities (e.g., increased blood pressure, hypertension, increased carotid intima-media thickness, higher heart rates, inferior contractility of the ventricles) in infants, toddlers, and adolescents who are HEU compared with those without HIV exposure, which may predict cardiovascular disease in adult life but requires further study [10,68-70]. HIV exposure is associated with a higher risk of altered lung function and chronic lung disease [71,72]. Additionally, adolescents and young adults who are HEU are four times more likely to develop reactive airway disease compared with individuals without HIV exposure [73]. Metabolically, in utero HIV exposure is associated with lower insulin sensitivity and metabolic abnormalities in infants who are HEU [26,74], which may predispose them to diabetes later in life. Risk of diabetes may be heightened if adolescents and young adults who are HEU concomitantly are overweight or obese [86]. In a small US cohort, obesity was >4 times more likely to occur in adolescents who are HEU compared with adolescents without HIV exposure [73]. Additionally, systematic reviews and a prospective cohort study reported high rates of psychiatric disorders, including depression, anxiety, and substance use, in children and adolescents who are HEU [12,67]. Whether parental factors are associated with morbidity requires dedicated assessment. 

These findings highlight the importance of early antenatal ART initiation to achieve sustained parental viral suppression, with parental risk-based consideration of breast/chestfeeding, screening protocols to identify infants, children, and adolescents at risk for poor health outcomes, and early interventions to mitigate risks.

Discussion

Interpreting results from studies on health outcomes among children who are HEU is challenging, as initial studies predominantly employed secondary data from cohorts established for other purposes [9,87], limiting comparisons with appropriate control groups and longitudinal observations. Socioeconomic factors and demographics were often not accounted for, further complicating analyses [6,7]. Some studies found no differences by HIV exposure, but may not have been adequately powered to detect clinically meaningful differences. Additionally, evolving ART guidelines from 2006 to 2015 led to variations in ARV exposure and duration between study populations [7]. Selection bias, high loss to follow-up, and lack of blinded analyses also may have resulted in flawed findings [6].

Many data gaps exist regarding health disparities observed in children who are HEU (Table 3). Several of these health disparities are interlinked (e.g., in utero HIV exposure is associated with low birth weight, which is associated with increased infant mortality) [7], highlighting the complexity of analyzing health and developmental disparities in persons who are HEU. Given the undeniable benefit of ART use from the time of diagnosis [88,89], it is not possible to disentangle associations between outcomes following in utero exposure to ARVs versus ARV postnatal prophylaxis. Although studies generally report that parental health and HIV control are associated with improved outcomes (Poster: Serghides, 2023) [11,32,81,84], the underlying mechanisms of health disparities most likely involve multiple intersecting factors, including timing of ART initiation, ART composition, parental viral load, the inflammatory environment, parental mental health, infant feeding practices, and other socioeconomic factors. Establishing a harmonized approach to control these potential factors will be important in future studies and enable data pooling across studies, increasing the potential to identify meaningful differences. As newer ARVs and treatment modalities are made available to pregnant and lactating persons with HIV, we must be equally positioned to assess both short-term (i.e., birth outcomes) and long-term impacts (i.e., academic achievement, cardiometabolic health) following fetal exposure. Fortunately, multiple ongoing real-world cohorts are assessing DTG exposure in children who are HEU, which will help address some of these data gaps, including the US Pediatric HIV/AIDS Cohort Study Surveillance Monitoring for ART Toxicities study, the Kenyan HOPE study, the Botswana FLOURISH study, the South African CHERISH study, and others [45,55,90,91].

**Table 3:** Summary of Key Data Gaps in Health Disparities Among Children Who Are HEU ART, antiretroviral therapy; DTG, dolutegravir; HEU, HIV-exposed but without HIV; INSTI, integrase strand transfer inhibitor.

Topic	Data gaps
Impact of various factors on health outcomes	How do HIV-related factors (e.g., ART exposure timing and composition, parental viral load), inflammation, and socioeconomic factors affect health disparities? Do health disparities observed in infants and young children persist as children age (through adolescence and young adulthood), and which factors primarily drive any long-term effects? What covariates are important for influencing the effects of HIV-related factors, inflammation, and socioeconomic factors on health outcomes? What is the impact of DTG and other second-generation INSTIs on health outcomes, including in the long term? What is the impact of sex differences on health outcomes?
Interventions and clinical management strategies	Which antimicrobial prophylaxis agents provide the best health outcomes, and what is the optimal timing of their administration after birth? What is the optimal parental and infant vaccination schedule for this population? What strategies can be used to scale up the use of interventions in the most affected areas? What should be included in a framework to facilitate long-term follow-up to monitor health outcomes in this population?

Assessing longer-term outcomes among adolescents and adults who are HEU will require a paradigm shift. Generally, once a child with in utero HIV exposure is no longer at risk of HIV acquisition, with documented negative HIV testing, key socioeconomic data and exposure history, including ARVs to which they are exposed, duration of exposure, and parental viral load during gestation are not tracked, making it nearly impossible to perform rigorous long-term research. Additionally, persons who are HEU must be aware of their exposure status, yet disclosure does not consistently occur for a variety of reasons [92]. Solutions to these challenges must be creatively addressed.

Our narrative review has several strengths, including the large number of articles included and the depth of data extraction. However, it also has limitations consistent with the narrative review approach. First, we incorporated conference proceedings; although these sources can offer timely insight into emerging findings, they are not peer-reviewed and should therefore be interpreted with caution. Second, although we summarized key statistical results from each study, a meta-analysis would have provided a more robust assessment of effects across studies. Additionally, we did not perform a formal risk-of-bias assessment, which would have allowed for a more systematic evaluation of study quality.

Many children who are HEU will thrive without the need for interventions. However, there is a critical need for validated screening tools capable of identifying children who are HEU at risk for poor outcomes, including poorer growth, suboptimal neurodevelopment, and infectious morbidity. Risk assessment tools will require integration into routine encounters with infants and children who are HEU. Tools must be easy to administer and ideally not require administration by already overextended healthcare clinicians.

## Conclusions

Increased availability of ART during pregnancy and breast/chestfeeding has substantially reduced pediatric HIV acquisition, an achievement worth celebrating. While efforts are ongoing to reduce HIV incidence among adults of childbearing potential, it is important to ensure that the population of children, adolescents, and young adults who are HEU are not only living without HIV but also achieving optimal health, development, and well-being outcomes. Well-designed studies are needed to continue to assess for health disparities and the underlying mechanisms of these disparities among children who are HEU. As mechanistic pathways are identified, clinical trials are needed to determine practical, impactful interventions. Trial results should inform health policies and programming. Importantly, health and educational systems will require adaptation to measure pragmatic outcomes upon implementation. In the maturing HIV epidemic, it is important to ensure that funding for rigorously conducted research around the health and well-being of children, adolescents, and young adults who are HEU exists. It is time to move beyond celebrating starting life without HIV to programming that ensures optimal outcomes into adulthood for children who are HEU.

## Appendices

Search strategy and article selection

This review was informed by a series of targeted literature reviews using PubMed for published manuscripts conducted through February 21, 2024 (search details below). Additionally, conference proceedings from January 2018 to April 12, 2023, were identified using Embase®. Articles were screened for relevance to the core topics of growth and development, neurodevelopment, immune health and function, and morbidity and mortality. Studies published January 1, 2012, or later were prioritised to focus on the contemporary evidence. To ensure a more comprehensive overview, we also included articles identified through the authors’ expert opinion, including publications and conference proceedings that became available after the database searches. Key background articles published before 2012 were included in some instances to provide important context.

Search strings

Search: Initial PubMed Search

String: ("HIV"[Title/Abstract] OR "HIV-1"[Title/Abstract] OR "immunodeficiency"[Title/Abstract]) AND ("adolescent"[Title/Abstract] OR "child*"[Title/Abstract] OR "infant"[Title/Abstract] OR "pediatric"[Title/Abstract]) AND ("HEU"[Title/Abstract] OR "exposed uninfected"[Title/Abstract] OR "CHEU"[Title/Abstract])

Search period: January 1, 2018, to February 21, 2024

Search: Updated PubMed Search (to Broaden Articles for Inclusion)

String: (("HIV"[Title/Abstract] OR "HIV-1"[Title/Abstract] OR "immunodeficiency"[Title/Abstract]) AND (“*HEU”[Title/Abstract] OR “*utero”[Title/Abstract] OR "child*"[Title/Abstract] OR "infant*"[Title/Abstract] OR “newborn*”[Title/Abstract] OR “neonat*”[Title/Abstract] OR “pediatric”[Title/Abstract] OR “adolescent”[Title/Abstract] OR “pregn*”[Title/Abstract] OR “postpartum”[Title/Abstract] OR (“birth”[Title/Abstract] NOT “sex”[Title/Abstract]) OR “maternal”[Title/Abstract]) AND (“dolutegravir” OR “DTG” OR “bictegravir” OR “BIC” OR “cabotegravir” OR “CAB” OR “elvitegravir” OR “EVG” OR “integrase”) NOT (“mouse”[Title/Abstract] or “zebrafish”[Title/Abstract] or (“pharmacokinetic”[Title/Abstract] AND “model*”[Title/Abstract])))

Search period: Any article published up to February 21, 2024

Search: Embase Conference Proceedings Search

String: ('hiv'/exp OR 'hiv' OR 'hiv-1'/exp OR 'hiv-1' OR 'immunodeficiency'/exp OR 'immunodeficiency') AND ('adolescent'/exp OR 'adolescent' OR 'child*' OR 'infant'/exp OR 'infant' OR 'pediatric'/exp OR 'pediatric') AND ('heu' OR 'exposed uninfected' OR (exposed AND uninfected) OR 'cheu') AND (2018-2023)/py

Search period: January 1, 2018, to April 12, 2023
